# *Ostrinia *revisited: Evidence for sex linkage in European Corn Borer *Ostrinia nubilalis *(Hubner) pheromone reception

**DOI:** 10.1186/1471-2148-10-285

**Published:** 2010-09-16

**Authors:** Shannon B Olsson, Subaharan Kesevan, Astrid T Groot, Teun Dekker, David G Heckel, Bill S Hansson

**Affiliations:** 1 Department of Evolutionary Neuroethology, Max Planck Institute for Chemical Ecology, Jena, 07745, Germany; 2 Central Plantations Crops Research Institute, 671124 Kasaragod, Kerala, India; 3 Department of Entomology, Max Planck Institute for Chemical Ecology, Jena, 07745, Germany; 4 Division of Chemical Ecology, Swedish University of Agricultural Sciences, PO Box 44, SE-230 53, Sweden

## Abstract

**Background:**

The European Corn Borer, *Ostrinia nubilalis *(Hubner), is a keystone model for studies on the evolution of sex pheromone diversity and its role in establishing reproductive isolation. This species consists of two sympatric races, each utilizing opposite isomers of the same compound as their major pheromone component. Female production and male response are congruent in each race, and males from each strain exhibit phenotypic differences in peripheral physiology. Both strains possess co-localized pheromone-sensitive olfactory sensory neurons characterized by a larger amplitude action potential (spike) responding to the major pheromone component, and a smaller spike amplitude cell responding to the minor component, i.e. the opposite isomer. These differences in amplitude correspond to differences in dendritic diameter between the two neurons. Previous studies showed that behavioral response to the pheromone blend was sex-linked, but spike amplitude response to pheromone components matched autosomal, not sex-linked inheritance.

**Results:**

As part of a larger study to finely map the loci responsible for pheromone communication in this species, we have reanalyzed peripheral physiology among parental, and first and second generation hybrids between the two pheromone strains using tungsten electrode electrophysiology. Our results reveal that differences in spike amplitude ratio between male pheromone-sensitive sensory neurons in *O. nubilalis *races are controlled, at least partially, by sex-linked genes that exhibit E-strain dominance.

**Conclusions:**

We propose that peripheral olfactory response in *O. nubilalis *may be affected both by autosomal and sex-linked genes exhibiting a cross-locus dominance effect, and suggest that the genetic basis for pheromone reception and response in the species is more closely linked than previously thought.

## Background

In recent years, sensory systems have received significant attention as catalysts to establish reproductive isolation between populations [1]. Chemical signals are perhaps the most ubiquitous sensory system, mediating behaviors between systems as diverse as gametes [2], and plants [3]. The evolution of pheromone diversity is an excellent resource to evaluate the role of sensory systems in speciation, both for its prevalence among several taxa and its definitive signal [4].

In insects, sex pheromones are typically blends of small numbers of volatile organic compounds (e.g. [5]). Minute changes in the ratio or identity of the blend can drastically alter the nature of the response. These characteristics result in both an extraordinary specificity in pheromone communication, as well as the potential for huge diversity in pheromone blends [4]. Sex pheromones thus provide an enticing evolutionary palette for the analysis of reproductive isolation and the development of species diversity.

Perhaps the most well studied system for assessing the role of pheromone diversity in reproductive isolation is the European Corn Borer (ECB; *Ostrinia nubilalis*). This species consists of two sympatric races, each utilizing opposite ratios of components in the otherwise similar pheromone blend. The "E-strain" uses a 99:1 ratio of *E:Z*-11-tetradecenyl acetate while the "Z-strain" uses a 3:97 ratio of the E:Z isomers [6-8]. Female production and male response are congruent in each of these races, although E-strain males have been shown to respond to a wider array of blend ratios than Z males [9].

In addition to behavioral response, males from both races can be distinguished by their peripheral physiology. Male *O. nubilalis *antennae possess pheromone-responsive trichoid sensilla containing 1-3 olfactory sensory neurons (OSNs; [10]). The most common pheromone sensilla contain at least two cells, one responding to the E isomer of the pheromone blend and the other responding to the Z [10-13]. The two pheromone strains can be distinguished by OSN action potential (spike) amplitude in response to the pheromone components. In the E-strain, the large amplitude cell responds to *E*-11-tetradecenyl acetate, the major component of this strain, while the smaller amplitude cell responds to the Z isomer. These amplitudes are reversed in the Z-strain [10-13]. This corresponds to roughly a 35/65% amplitude ratio of Z:E responsive cells in E-strain males, and the reverse in the Z-strain [11]. Morphological studies show that these differences in amplitude are correlated with a variation in the diameter of the neuronal dendrites housing receptors for each isomer [10,12]. Hybrids exhibit an intermediate phenotype I, with a 50/50% ratio, and the non-overlapping response ratios allowed individuals to be classified into three distinct phenotypes, E, I, and Z. These phenotypes appeared to be determined by a single autosomal locus, as they occured in the expected Mendelian ratio 1:2:1 in F_2 _progeny and 1:1 ratios in both backcrosses [11].

In the first attempt to compare the genetic basis of pheromone communication at the level of pheromone production, male behavioral response, and male peripheral physiology, Roelofs et al. [14] scored all of these traits in the same set of crosses. Because females are the heterogametic sex in moths, different predictions result from autosomal vs sex-linked inheritance, depending on the type of cross (see Table 1 for predictions from crosses used in this study). They confirmed the autosomal hypothesis for sensillar phenotype on the basis of F_2 _and backcross generations. However, they found that male behavioral flight response was sex-linked [14]. Female pheromone production was also determined by an autosomal gene, which was subsequently shown to be unlinked to the male antennal phenotype gene [15]. The loci for female pheromone production (*pher*) and male flight response (*resp*), were later mapped onto an autosome and sex chromosome, respectively, using AFLP (amplified fragment length polymorphism) and microsatellite markers [16,17]. Recently, the gene underlying the difference in female pheromone production has been identified [18]

**Table 1:** Results of ANOVA tests for single sensillum response amplitude ratios among *O. nubilalis *parents and crosses

	*** ANOVA	Sex-linked prediction	Autosomal prediction	x	N	SN	Parent		Hybrid		Female-informative Backcross				Male-informative Backcross	
**Code**							**1**	**2**	**3**	**4**	**5**	**6**	**7**	**8**	**9**	**10**

	**Cross**						**E**	**Z**	**ExZ**	**ZxE**	**EZxE**	**EZxZ**	**ZExE**	**ZExZ**	**ExZE**	**ZxZE**

**1**	**E**	X^E^X^E^	A^E^A^E^	1.53	6	23		**				***				*
		
**2**	**Z**	X^Z^X^Z^	A^Z^A^Z^	-1.24	10	43			*	*	**		***	**		
			
**3**	**ExZ**	X^Z^X^E^	A^Z^A^E^	0.45	13	54						**				
				
**4**	**ZxE**	X^Z^X^E^	A^Z^A^E^	0.62	13	56						***				
					
**5**	**EZxE**	X^Z^X^E^	A^Z^A^E^+A^E^A^E^	1.33	21	69						***			*	**
						
**6**	**EZxZ**	X^Z^X^Z^	A^Z^A^Z^+A^Z^A^E^	-1.98	18	59							***	***	*	*
							
**7**	**ZExE**	X^E^X^E^	A^Z^A^E^+A^E^A^E^	1.57	14	39									*	**
								
**8**	**ZExZ**	X^Z^X^E^	A^Z^A^Z^+A^Z^A^E^	0.96	21	80										*
									
**9**	**ExZE**	X^Z^X^E ^+X^E^X^E^	A^Z^A^E^+A^E^A^E^	-0.20	12	58										
										
**10**	**ZxZE**	X^Z^X^Z ^+X^Z^X^E^	A^Z^A^Z^+A^Z^A^E^	-0.51	27	111										
										
	**Total**				155	592										

(ANOVA *p *< = 0.001). Asterisks for comparisons indicate results of Post Hoc LSD Tests for Individual: *p < = 0.05 **p <= 0.01 ***p <= 0.001. x lists the mean population E/Z amplitude ratio, N represents number of individuals tested, and SN refers to number of sensilla contained per group. Sensillum ratios were averaged within each individual and are shown in Figure 2. For expected genotypes, ^E^: E pheromone, ^Z^: Z pheromone.

The apparent independence between the inheritance of flight behavior and male peripheral response to the pheromone blend was surprising. As with male behavior, all F_1 _hybrid individuals exhibited intermediate phenotypes with equal spike amplitude OSNs responding to *E *or *Z*-11-tetradecenyl acetate. However, second generation phenotypes corresponded to autosomal, not sex-linked loci [11,14]. A later study coupled behavioral and electrophysiological analyses of second generation hybrids to reveal individuals with the peripheral physiology of one strain, and the behavior of another, emphasizing the lack of direct linkage between pheromone behavior and peripheral physiology [13]. A more recent study performed antennal disc transplants between strains to produce males with the antenna of one strain and the CNS of another [19]. These individuals always exhibited the behavior of the recipient strain, even if the transplanted antennae originated from the opposite strain. These results implied that the divergence in behavior between E- and Z-strains of ECB stems from changes at central, not peripheral levels.

An initial study at the first level of olfactory processing in the ECB brain, the antennal lobe, showed that a greater proportion of antennal lobe neurons responded to the major component in each strain [20]. Offspring of the female-informative backcross of an EZ female with a Z male (in short, EZxZ; the first letter always denotes the female) also possessed more Z-responsive cells, reflecting the sex-linkage of the behavioral phenotype. However, ZxE F_1 _hybrids showed a dominance of E-responsive cells similar to E-strain parents, which seemed to contradict the intermediate behavioral response of F_1 _hybrids in the 1987 study [14]. A recent neuroanatomical study of central projection neurons and peripheral sensory neurons also observed that both parent strain males possessed morphologically identical antennal lobes [21]. The CNS (central nervous system) of both strains contained a pheromone response center, known as the macroglomerular complex, with two highly convoluted bundles of neuropil (glomeruli), and a third, smaller glomerulus. The two larger glomeruli expressed a reversed topology, with the major component always targeting the medial glomerulus, and the minor component the lateral. The authors therefore suggested that the single shift in behavioral response could be attributed to peripheral, not central, pathways [21].

Whereas CNS studies point to the periphery as the source for behavioral divergence, physiological studies of OSNs suggest that changes in central pathways are responsible for the behavioral alterations. This reveals an apparent paradox between physiology and behavior in males of this species. We therefore reanalyzed peripheral physiology among parents, and first and second generation hybrids between the two pheromone strains of ECB. In contrast to the original studies of this trait [11,14], we found that the sensillar phenotype is controlled, at least partially, by sex-linked genes that exhibit partial E-strain dominance. Comparison to a more recent study also indicates both sex-linked and autosomal genes segregating in the same cross [13]. Our new findings on the inheritance of peripheral physiology in this species suggest that a significant revision of the genetic basis for peripheral olfactory response in *Ostrinia *is in order, and raises the possibility that at least one of the genes affecting sensillar response could be the same as, or linked to, sex-linked genes affecting male behavioral choice.

## Results

### Comparison of Parent, F_1 _Hybrid, and Backcross OSN Responses

Olfactory sensilla (n = 592) from 155 individuals across 10 strains and crosses of ECB were used for electrophysiological analysis. An average of 3.84 sensilla were recorded from each individual.

**Figure 1:**
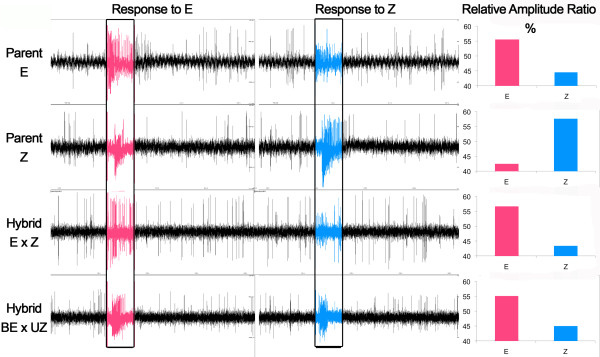
**Sample responses of pheromone sensitive sensilla for parent and F_1 _hybrids of *O . nubilalis***. Traces are scaled to 1.2 mV and approximately 3 s. Bars at the base of all traces show the location of the 0.5 s stimulus pulse. Left traces show response to the E pheromone isomer and right traces show response to the Z. Bar graphs on right show the relative amplitude ratio % for the first five spikes in the traces shown.

**Figure 2:**
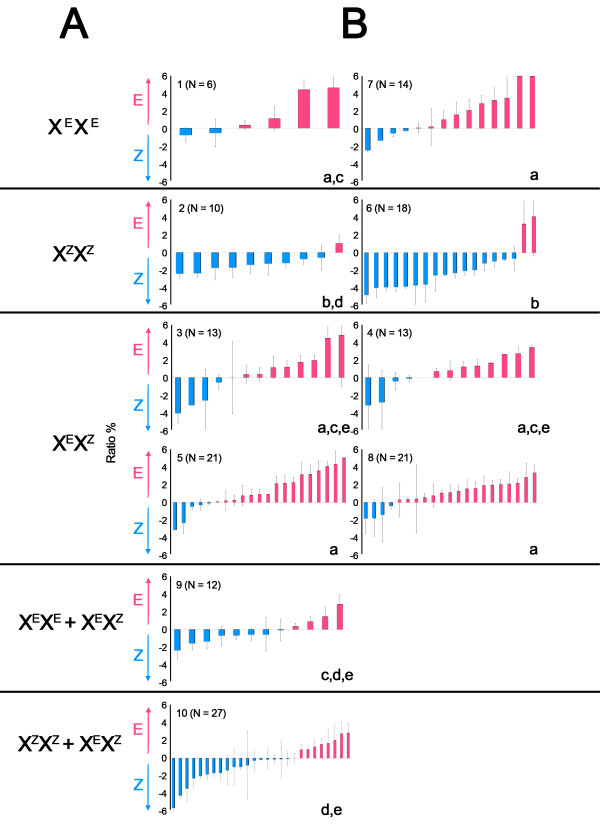
**Average single sensillum amplitude ratios for all individuals in each *O. nubilalis *strain or cross**. A) Populations are ordered according to predicted genotype assuming sex linkage of the peripheral amplitude trait (Table 1). B) Individuals are arranged from lowest to highest average ratio for each strain or cross. Ratios compare the spike amplitude of the response to the E pheromone isomer and the Z, and are presented at ***x***-50% so that individuals with equal average amplitude responses to both isomers lie at 0%. Individuals above 0 have a higher average amplitude response to the E isomer while individuals below 0 have a higher average response to Z. Letters in the corner of each graph show least significant difference groupings (***p***> 0.05) according to post-hoc LSD tests for individual (Table 1). Populations with different letters are significantly different according to LSD tests (***p ***< = 0.05), and populations with multiple letters are common to more than one group.

Figure 1 illustrates sample responses from parent and F_1 _hybrid sensilla to both isomers. Note that a clear distinction in spike amplitude can be made between OSNs in both parent and hybrid examples. Figure 2 shows mean E/Z response ratios for all recorded sensilla from each parent and hybrid individual. Individuals are arranged from lowest to highest ratio, in a manner similar to Cossé et al [13]. Thus, the leftmost individuals exhibited responses with the highest relative Z amplitude spikes (action potentials), whereas the rightmost had the highest relative E amplitudes. The graphs illustrate variation in spike amplitude ratio both within and between groups. Notably, not all E parent strain sensilla contained large amplitude OSNs responding to the E isomer, and not all Z-strain sensilla contained large amplitude OSNs responding to the Z isomer; there is an overlap in the distribution of responses of the two strains.. Additional file 1 shows a similar distribution when single sensilla are compared irrespective of individual. This shows that the variation was consistent across individuals. Statistical tests of sensillar variation (not shown) also produced similar results to the per individual tests described below.

When spike amplitude ratios were compared per individual, several differences among the crosses could be found (Table 1). Specifically, the E parent strain (population 1) possessed more sensilla containing large amplitude E responding cells than the Z parent strain (2; *p *< = 0.01). Both hybrid crosses (populations 3 and 4) also had more sensilla with large amplitude E cells than the parent Z-strain (2; *p *< = 0.05), but were not different from the parent E-strain (1) nor from each other. In the female-informative backcross generation (defined here as a hybrid female crossed to a parent strain male), both EZxE (5) and ZExZ (8) crosses as well as the ZExE (7) cross possessed sensilla with more large amplitude E cells than the parent Z-strain (2; *p *< = 0.01). These backcrosses were not significantly different from the parent E-strain (1). Conversely, the EZxZ (6) backcross possessed more sensilla containing large amplitude Z cells than the E-strain (1; *p *< = 0.001) as well as the other 3 female-informative backcrosses (5,7,8; *p *< = 0.001), yet was not different from the parent Z-strain (2). The male-informative ZxZE backcross (10; defined here as a parent strain female crossed to a hybrid male) contained more sensilla with large amplitude Z cells than the parent E-strain (1; *p *< = 0.05), but was not different from the Z-strain (2) or either F_1 _hybrid cross (3 and 4). Finally, the ExZE (9) backcross was not different from any parent or F_1 _hybrid group.

### Comparison to Genetic Models

We now examine the patterns of significant differences between the numbered crosses in Table 1 and Figure 2 in light of the genetic models. The autosomal model predicts that the distributions within crosses numbered 6 (EZxZ), 8 (ZExZ), and 10 (ZxZE) should be equal, as all three backcrosses to Z have the same predicted genotypes (1:1 A^Z^A^Z ^and A^Z^A^E^). This prediction does not depend on the degree of dominance of the A^E ^allele. However, the comparisons of 6 vs 10 and 8 vs 10 are significantly different (*p *< = 0.05, ANOVA, Table 1) and 6 vs 8 are highly significantly different from each other (*p *< = 0.001). The autosomal model also predicts that the distributions within the three crosses 5 (EZxE), 7 (ZExE) and 9 (ExZE) should be the same, as all three backcrosses to E have the genotypes A^Z^A^E ^and A^E^A^E ^in a 1:1 ratio. This prediction also does not depend on the degree of dominance of the A^E ^allele. However, although the comparison of 5 vs 7 is nonsignificant, 5 vs 9 and 7 vs 9 are significantly different (*p *< = 0.05). Thus, five out of six predictions of the autosomal model can be rejected with our data.

The sex-linked model makes different predictions about which distributions should be the same: 1 = 7 (both pure E and ZExE having the predicted genotype X^E^X^E^), 3 = 4 = 5 = 8 (all F_1 _and some backcrosses with the predicted genotype X^Z^X^E^), and 2 = 6 (pure Z and EZxZ both predicted to be X^Z^X^Z^). These sex-linked predictions are also independent of the degree of dominance of the X^E ^allele. None of these comparisons are significantly different as shown by Table 1 and Figure 2, and thus we cannot reject these predictions of the sex-linked model.

In most cases where the autosomal model fails, the sex-linked model gives a better qualitative prediction (Figure 2, Additional file 2). For example, in contrast to the autosomal prediction of 6 = 8 = 10, the sex-linked model predicts 6 < 10 < 8 because of the increasing numbers of X^E ^alleles expected in the progeny, and this qualitative prediction conforms to the data. However, the autosomal prediction of 5 = 7 = 9 is the same as the sex-linked model if the X^E ^allele is completely dominant; if not, the sex-linked model predicts 5 < 9 < 7, and the order according to the data is 9 < 5 < 7. Cross type 9 has the same predicted genotype configuration under both models, and is inconsistent with both models due to a lower score placing it closer than expected to the ZZ homozygote. Thus, except for one cross (9) that is inconsistent with both models, the sex-linked model more accurately predicts the rank order of different cross types than the autosomal model.

**Figure 3:**
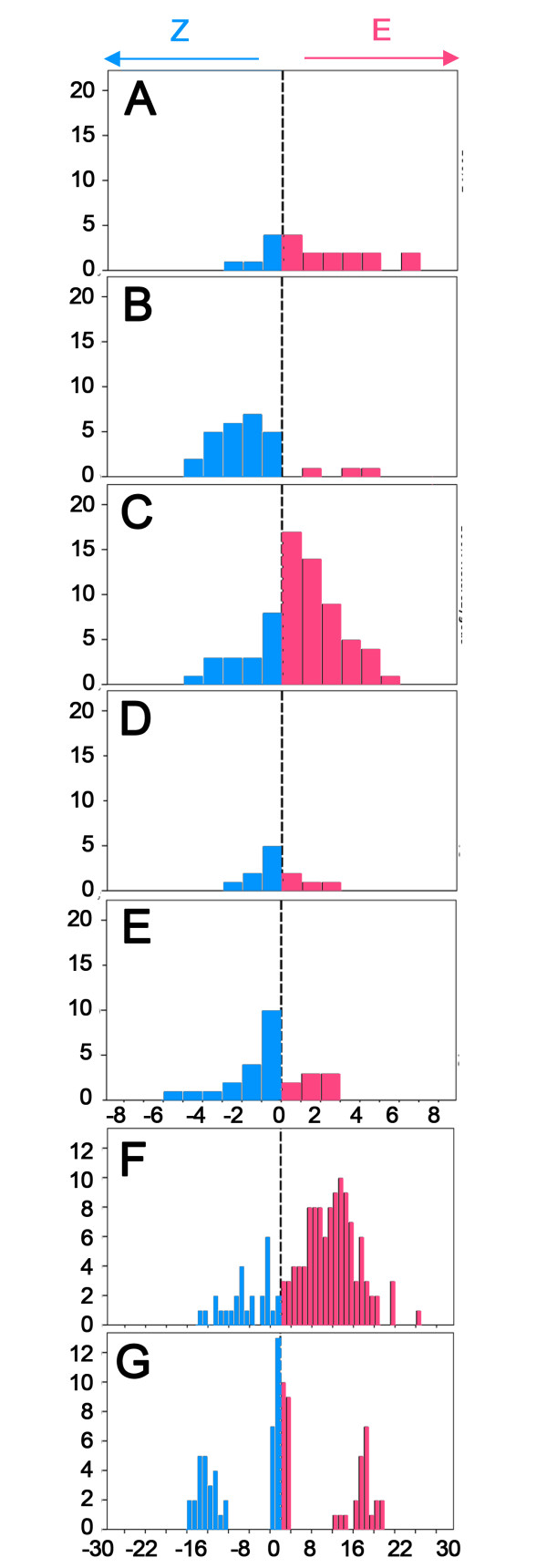
**Average amplitude ratio histograms for *O. nubilalis *populations**. Frequency distributions of average sensillum amplitude ratios combined from crosses shown in Figure 2. Each histogram depicts the sum of all individuals expected to carry specific genotypes according to the sex-linked hypothesis (see Figure 2 for separate populations): A) X^E^X^E ^B) X^Z^X^Z ^C) X^E^X^Z ^D) X^E^X^E ^+ X^E^X^Z ^and E) X^Z^X^Z ^+ X^E^X^Z^. Bottom figures show previously published data from F) Cossé et al., 1995 [13] and G) Hansson et al., 1987 [11]. Note the different x-axis scale in histograms F) and G).

The model of a sex-linked locus for average sensillar response ratios fits our data better than the single autosomal codominant gene model found by earlier studies [11]. Pooling all crosses with the same predicted genotypes under the sex-linked model yields the overall distributions characteristic of X^E^X^E ^homozygotes (Figure 3A; x = 1.56), X^Z^X^Z ^homozygotes (3B; x = -1.72), and X^Z^X^E ^heterozygotes (3C; x = 0.91). These distributions have extensive overlap but different means. The mean of heterozygotes is closer to that of X^E^X^E ^homozygotes, indicating partial dominance of the X^E ^allele on this particular phenotype.

### Comparison of European and American Populations

In addition to the 10 strains and crosses obtained from European locations, a small subset of bivoltine E- (BE) and univoltine Z- (UZ) strains of ECB were obtained from American colonies to compare pheromone response profiles between continents. These tests were also performed to control for any conflicting results with previous studies that used only American strains. We recorded from a total of 66 sensilla from 15 bivoltine E X univoltine Z males.

**Figure 4:**
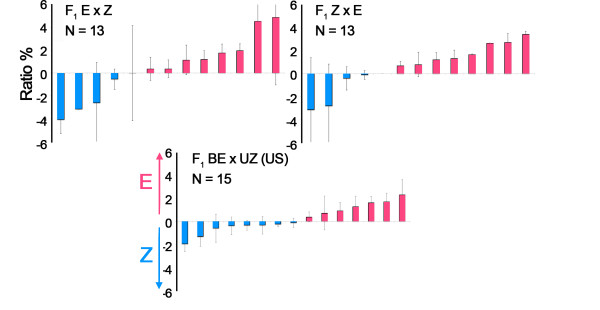
**Average single sensillum amplitude ratios for individuals in each *O. nubilalis *F_1 _hybrid cross**. Individuals from European (top) and American (bottom) origin crosses are arranged from lowest to highest average ratio for each group as in Figure 2.

Figure 4 provides a comparison of E/Z response ratios for all individuals from both F_1 _hybrid European strains as well as the American ExZ hybrids. Again, the graphs show a variety of response ratios both within and between groups. However, the American BExUZ population was not different from either European hybrid population (one way ANOVA *p *= 0.881), but was different from the parent strains (Kruskal-Wallis *p *< = 0.05). Post hoc tests showed that the American hybrid cross possessed more sensilla containing large amplitude E cells than the European parent Z-strain (Mann-Whitney *p *< = 0.01), but was not different from the European parent E-strain. Thus, the distribution of response amplitudes from both European and American F_1 _hybrids were similar to each other (Levene's Homogeneity of Variance *p *= 0.125 for the three hybrid populations).

## Discussion

The results of this study confirm that the two pheromone strains of *O. nubilalis *exhibit differences in action potential amplitudes between pheromone responsive cells. The E-strain possessed a significantly higher proportion of sensilla in which the large amplitude OSN responded to their major component, *E*-11-tetradecenyl acetate, and a smaller, co-localized cell responded to the Z isomer. Conversely, the Z-strain possessed a significantly higher proportion of sensilla in which the large amplitude OSN responded to their major component, *Z*-11-tetradecenyl acetate, and a smaller cell responded to the E isomer. These two strains were significantly different with respect to E:Z OSN amplitude ratio (*p *< = 0.01).

However, the antennal responses of hybrid offspring contrasted sharply with previous studies [11,14]. The current study found that reciprocal F_1 _hybrids between the two strains possessed large amplitude OSNs responding to *E*-11-tetradecenyl acetate, and smaller OSNs responding to the Z isomer, similar to the E parent strain. Additionally, antennae from three of the four female-informative backcrosses (ZExE, ZExZ, and EZxE) contained sensilla with large amplitude cells responding to the E isomer. Conversely, the EZxZ backcross possessed sensilla with large amplitude cells responding to the Z isomer.

The results of this study do not match the patterns expected for autosomal inheritance (Table 1). Rather, the current results suggest that peripheral olfactory response (i.e., spike amplitude) to pheromone components in *O. nubilalis *is controlled, at least partially, by sex-linked genes that exhibit E-strain partial dominance. If one assumes sex-linked partial E-dominance, then F_1 _hybrid individuals containing heterozygous alleles from both strains should be more similar to the E-strain. Additionally, all female-informative backcrosses should be more similar to the E-strain, except for an EZxZ cross (shown in Table 1), which contains homozygous sex-linked alleles from the Z-strain, and will resemble the Z parents. This pattern is precisely what is shown in Table 1 and Figures 2 and 3.

Although these results contrast with prior electrophysiological analyses [11,14], they provide a key to resolving some standing conflicts between behavioral and physiological studies. Namely, the behavioral response to pheromone blends in ECB has been shown to be sex-linked [14], but previous studies found that autosomally-controlled peripheral response did not correspond to behavior [13], or pheromone production [15]. Current results show that peripheral response can also be affected by variation in a gene or genes on the sex chromosome, which matches the sex-linkage found for behavior in previous studies (we did not directly measure male behavioral response in our crosses). Additionally, electrophysiological recordings from single antennal lobe neurons showed a prevalence of E-isomer responsive cells in both E-strain and F_1 _hybrids, and more Z-responsive cells in Z-strain and EZxZ backcross individuals [20]. A report published in this volume also shows that glomerular innervation of projection neurons corresponds to E-dominant sex linkage [22]. These central studies match the peripheral results here.

Perhaps the most significant dilemma is why current results differ from previous genetic and physiological studies of the Corn Borer antenna. Present results are, in fact, largely coherent with previous studies in terms of overall differences between E- and Z-strain amplitudes. However, we find differences in amplitude among F_1 _hybrids and backcrosses that were previously unobserved. Initially, it was proposed that differences could be due to the strains used. In previous studies, American strains of *O. nubilalis *were obtained mainly from New York populations. In this study, moths were of European origin, from Hungary and Slovenia. Nevertheless, a comparison of the two populations (Figure 3), show no significant differences in amplitude ratio.

However, previous studies also differ in aspects of data sampling, variability, and averaging of several responses per individual. The first study [11] revealed discrete, non-overlapping distributions that were assigned to three phenotypes, which we have combined in Figure 3G. These values are a subset of the entire variability exhibited by those crosses as some sensilla were assigned to a phenotype by visual inspection of the spikes rather than on the basis of recordings. Similarly, the companion study comparing electrophysiological phenotypes, flight behavior and female pheromone production [14] reported phenotype frequencies only and did not show the distribution of recorded E/Z spike ratios. In both of these studies, only a single sensillum was recorded per individual. Thus, the biological variation within phenotypic classes may have been under-represented by the sampling techniques employed. However, the differences between phenotypic classes were very great and the assignment to classes was probably not affected by the data sampling methods used.

A principal reason for discrepancies with previous results may be that microscopy, electronic and computing equipment have developed considerably in the past 20 years, and thus allow for much finer delineation of spike heights than the original 1987 study [11], particularly among F_1 _hybrids. Additionally, previous studies of ECB have all utilized the "cut tip" technique [23,24], where single sensilla are cut at the distal tip, and exposed OSN dendrites are contacted by covering the cut hair with a saline-filled glass electrode. In this study, a finely sharpened tungsten electrode was used to penetrate the base of each sensillum and record the activity of OSNs contained therein. With the tungsten recording technique, OSN activity is recorded much closer to the source of action potential generation, the cell body, and may measure more subtle differences in spike amplitude than the apical cut tip technique. Morphological analyses found that OSN dendrites tapered distally [12], which could make differences in diameter and amplitude more difficult to discern near the tip of the sensillum. Morphological studies also showed that although dendrite diameters were more similar in F_1 _hybrids, a difference in diameter could still be observed [12], although differences in amplitude could not be measured. *Ostrinia *is also notoriously difficult for peripheral electrophysiology studies. Even so, Hansson et al. [12] notes that relationships in spike amplitude have remained the same in studies of *Tricoplusia ni *and the turnip moth, *Agrotis segetum*, despite the technique used (see [25-28] in [12]).

Interestingly, the results of Cossé et al. [13] could also be re-evaluated given the current data. This previous study noted a mismatch between antennal physiology and behavior in F_2 _hybrids from an original Z-strain female and E-strain male cross. In that study, each male was first tested in a flight tunnel bioassay and classified as an E-responder, a hybrid-responder, or non-responder (a fourth class responding to both E and Z was omitted from the analysis). Each male was subsequently subjected to electrophysiological analysis using the cut-sensillum technique and classified as E-type, I-type, or Z-type based on relative spike amplitudes in response to antennal stimulation by the two pheromone components. The main conclusion of the authors was that all three antennal types were found in each of the three response types, suggesting that a male's behavioral response in the flight tunnel was independent of whether he carried an E-type, I-type, or Z-type antenna [13].

Combining all of the data reported on spike ratios in the F_2 _study [13] results in the histogram depicted in Figure 3F. The ratios range over the same magnitudes as in the earlier study [11], but discrete phenotypic classes are no longer recognizable. The authors therefore used two cutoff points based on the previous study and their own measurements of Z, E, and hybrid F_1 _populations to assign F_2 _males to Z, E, or I (hybrid) types. Under autosomal inheritance, the physiology of the F_2 _males should exhibit a 1:2:1 ratio between Z, hybrid, and E-type males. However, the observed numbers (20:41:77) deviate strongly from this prediction (χ^2 ^= 26.5, P < 0.0001), being much closer to 1:2:5 (χ^2 ^= 1.4, P < 0.5). The authors could find no obvious explanation for this deviation, but the large excess of E types could be explained by a second, sex-linked locus with a dominant E-strain allele. Under this hypothesis, because of the direction of the cross, half of the F_2 _males would be homozygous X^E^X^E ^for the E-strain allele, which shifts the autosomal gene distribution to the right and produces an E-type antenna in all but the leftmost A^Z^A^Z ^individuals. The other half of the F_2 _progeny would carry one Z-strain and one E-strain allele at the sex-linked locus (X^Z^X^E^), producing a much smaller rightward shift. Among these heterozygotes, the genotype at the autosomal locus would determine antennal type in the expected 1:2:1 ratio. Summing these two groups of males produces a 1:2:5 expected ratio overall. The overall distribution of the F_2 _population in Figure 3F resembles a superposition of the variation due to the sex-linked gene in Figure 3A and 3B on to the variation due to the autosomal gene in Figure 3G. Thus, the inheritance of antennal type in these F_2 _crosses cannot be explained by the classical model of a single autosomal locus, but instead requires a strong bias towards E-expression that could be provided by a second, sex-linked locus segregating 1:1 in the males. Under this hypothesis, the partial dominance of the sex-linked E allele is epistatic to the autosomal locus in determining the antennal type, which is a type of cross-locus dominance effect.

An additional possibility to explain the different magnitudes of sex-linked control on sensillar response in the different studies could be variable penetrance of a sex-linked locus. We cannot formally exclude this hypothesis with the data at hand, but it could be tested if genetic markers linked to the autosomal gene segregating in the earlier studies (11, 14) and sex-linked markers linked to the *resp *locus were both followed in the same set of crosses. So far, the former gene has not yet been localized on the genetic map along with *pher *and *resp *(15).

The source for this proposed epistatic effect could relate to the nature of the peripheral response. In the phenotype for spike amplitude response, there are two factors at play. The first is the amplitude of the action potential. This is likely due to the diameter of the dendritic shaft housing the olfactory receptors [10,12]. In each parent strain, receptors for the major pheromone component may be housed in the largest OSN in each sensillum, producing large amplitude spikes responding to the major component. The second factor is the olfactory receptor (OR) itself. Although the neuron transmits the electrical information to higher brain centers, ORs confer the specificity and response pattern of the OSN [29]. Antennal lobe neuroanatomy suggests that parent strains have "swapped" ORs between the two pheromone OSNs, but targeting of these OSNs remains the same [21]. Baker [30] further notes that OR swapping supports the theory of olfactory antagonistic balance, which has been suggested as a source for evolutionary divergence in these strains. It is entirely possible that OSN morphology and OR gene targeting are dictated by separate genes; one autosomal, and one sex-linked. Interestingly, two other sex-linked genes also correspond to differences between the strains. One gene, *Pdd*, corresponds to changes in pupal diapause between American strains [17]. The other gene, *Tpi*, codes for the enzyme triose phosphate isomerase [31]. Dopman et al. [17] suggests that *Tpi *allozyme frequency differences between E- and Z- strains might be due to linkage relationships to *resp *and *Pdd*. It is tempting to suggest that an E-dominant gene coding aspects of the peripheral response also lies somewhere in the vicinity these sex-linked genes, or may, in fact, be the *resp *gene itself. If the latter, additional explanations would be required to resolve the codominant effect of *resp *on male behavioral response and its semi-dominant effect on sensillar phenotype. We did not measure male behavioral response in this study; but behavioral mapping of the *resp *gene is currently under way in another set of crosses.

Notably, natural preferences in male *O. nubilalis *also vary, and male pheromone response at higher concentrations extends beyond the ratios produced by females [9]. In particular, the E-strain was found less specific to the 99:1 E:Z pheromone blend produced by females, and responded to a broad range of ratios. F_1 _hybrid males also responded to a range of blend ratios beyond the 65:35 E:Z blend produced by hybrid females [9]. Establishing whether the sex-linked gene affecting antennal type is the same as the sex-linked gene affecting flight response (*resp*) would require additional fine-scale mapping, which has not yet been attempted.

Nevertheless, epistatic effects cannot entirely account for the extent of variation both within and between individuals in this study. First, penetration of the sensillum cuticle could have produced some sampling error in spike size depending on proximity to the OSNs recorded. If the tungsten recording electrode was closer to one of the OSNs, it could potentially register a larger spike size for that cell. Because trichoid sensilla were chosen at random on the antenna, the choice of sensillum and location of the recording electrode in the sensillum also differed between trials. Additionally, a recent study suggests there is cross-reactivity between pheromone-responsive cells on the ECB antenna [32]. In that study, large spiking OSNs from the E-strain responded to both isomers. The study also notes that ECB is highly susceptible to neuronal adaptation, and broad selectivity could be erased if the major pheromone component was presented first. In the current study, pheromone components were presented randomly multiple times for each recorded sensillum. The first five responding spikes were then averaged regardless of spike size to avoid any bias in spike selection. It is possible that due to cross reactivity we inadvertently selected responses from both large and small spiking cells in a single trial, thus altering the spike size ratio for some cells. In any event, this variation proved consistent both within and between individuals (compare Figure 2 and Additional file 1), and statistical comparisons were coherent whether performed on a per-individual or per-sensillum basis.

## Conclusions

Our results indicate that the inheritance of peripheral olfactory response among male *O. nubilalis *is mediated by a more complex genetic pathway than previously thought. Both behavioral and electrophysiological response to pheromone components appears to be coded by sex-linked genes, and single or closely-linked genes coding for differences in OSN action potential amplitude exhibit partial dominance for the E-allele(s). We further propose that the sex-linked E allele is epistatic to an autosomal locus in determining the antennal type, which exhibits a type of cross-locus dominance effect. Previous studies on the autosomal inheritance of antennal type suggested that it was functionally independent of behavioral response, but inheritance of behavioral and physiological response on the same chromosome indicates the possibility for a mechanistic or genetic linkage. Further studies, including in situ expression of pheromone OR genes on the antenna, can elucidate if the *resp *gene coding for changes in pheromone behavior coordinates with alterations in OR expression between different pheromone-sensitive cells. Genetic analyses may also determine the heritable basis for dendrite diameter that leads to differences in OSN response amplitude in this species. In any case, *O. nubilalis *continues to provide a rich repository for studies on the evolution of pheromone communication, and the naissance of new species in general.

## Methods

### *Ostrinia *Origins and Rearing Conditions

Parent E and Z *O. nubilalis *strains were obtained as larvae and pupae from Z. Karpati, Swedish University of Agricultural Sciences (SLU) (Alnarp, Sweden). The Z-strain was derived from a colony established from a cornfield collection in Kéty town, county of Tolna, Hungary in 2004. The E-strain colony was established from a 2005 collection of larvae from maize stems by Smiljana Tomse (Agriculture and Forestry Institute, Novo Mesto, Slovenia). Larvae were reared on an artificial wheatgerm-based diet [33], and adults fed a diet of 10% honey water. All animals were maintained at 27°C and 70% humidity at a 16:8 L:D cycle. American strains of bivoltine E and univoltine Z *O. nubilalis *were kindly provided by Dr. Charles E. Linn, Jr., New York State Agricultural Experiment Station, Cornell University, Ithaca, NY. Individuals were shipped as pupae and reared as described above.

Single pair matings were set up, hybridizing Z females with E males (ZE) or vice versa (EZ). The families that produced most offspring were used for backcrossing. In single-pair matings, individual female F_1 _hybrids were backcrossed to E or Z males to produce the female-informative backcross families, while male F_1 _(ZE) hybrids were backcrossed to either parental strain to produce the male-informative backcross families. Families that produced most offspring were used for physiological analyzes. Adult males were used for electrophysiological analyses between 1-4 days of age.

### Chemical Stimuli

*E *and *Z*-11-tetradecenyl acetate were obtained from Pherobank (Wageningen, the Netherlands). Stock solutions (1 μg/μl) of individual key volatiles were prepared in hexane (Fluka Analytical) and 10 μl pipetted onto filter paper in disposable Pasteur pipettes. Blank stimuli containing 10 μl hexane and dilutions of each compound at 1 ng/μl, 10 ng/μl, 100 ng/μl were also prepared.

### Single Sensillum Electrophysiological Analysis

Adult male *O. nubilalis *were confined in the tapered, cut tip of a 100 μl disposable pipette with only their heads and antennae protruding. The body of the insect was immobilized with dental wax and a tungsten or silver wire inserted into the abdomen and thorax to serve as a ground electrode. The insect was then placed dorsally on a small piece of wax and its antennae immobilized on a coverslip covered with double stick tape. A sharpened glass pipette was placed on the first segments of the antenna near the scape to further immobilize the antenna. An electrolytically sharpened tungsten electrode was used to establish contact with pheromone sensitive olfactory sensory neurons (OSNs). The recording electrode was positioned at the base of a sensillum using an Olympus BX-51 microscope with 1000× magnification and a 3-D motorized micromanipulator system (Luigs-Neumann, Ratingen, Germany). In most cases, sensilla from the first 10 segments of the left antenna were used for recording. Pheromone-responsive trichoid sensilla were localized by morphology and response to both pheromone isomers.

Charcoal-filtered and humidified air passed over the antenna from a stimulus air controller at approximately 1 l/min (Syntech, CS-5, Hilversum). The constant flow included both a continuous (0.5 l/min) and a complimentary (0.5 l/min) air stream. Air passed through an aluminum tube protruding approximately 10 mm from the antenna. Stimulation was performed by inserting the tip of the test pipette into a hole in the metal tube, approximately 3 cm before the outlet. The test pipette was connected to the stimulus air controller, which generated air puffs (0.5 l/min during 0.5 s) through the pipette and replaced the complimentary air stream during that time period.

The analog signal originating from the OSNs was amplified (10×) (Syntech Universal AC/DC Probe, Hilversum), sampled (10667.0 samples/s) and filtered (100 Hz - 3000 Hz with 50/60 Hz suppression) via USB-IDAC connection to a computer (Syntech, Hilversum). Action potentials were extracted as digital spikes from the analog signal according to top-top amplitudes using Syntech Auto Spike 32 software. Differences in amplitude between co-localized OSNs were measured in mV for each recording based upon signal output.

Contacted OSNs were presented with 1 μg stimulus loadings of both pheromone isomers and the blank in random order. Stimuli were presented a total of three times. In many cases, adaptation-disadaptation trials were performed at the end of each recording period using 1 and 10 μg loadings of each isomer.

### Data Analysis

Analysis of single sensillum responses was based on Hansson et al. [11] and Roelofs et al. [14]. For each 10 s recording period, the amplitudes in mV of the first 5 OSN spikes (action potentials) after stimulation were measured and averaged. To compensate for the stimulus delay created by the distance between the outlet and the stimulus controller, spikes were measured beginning 150 ms after stimulus onset.

For each recorded sensillum, the average OSN spike amplitude 150 ms after stimulus onset was averaged across all stimulus replicates. These averages were then converted into ratio % [11] as follows to determine the relative amplitude of the E response to the Z:

$$\frac{\text{Average amplitude of E spikes (mV)}}{\text{(Average amplitude of E spikes + Average amplitude of Z spikes)}}\times 100$$

For statistical analysis, these ratios were compared across all strains and crosses by analysis of variance (ANOVA) tests using SPSS v.17 software. Pairwise post-hoc comparisons between two groups were performed using least significant difference (LSD) tests. Levene's test for homogeneity of variance showed that the American hybrid population and European parent populations together did not exhibit equal variances for parametric testing (*p *< = 0.05). Thus, a non-parametric Kruskal-Wallis test was used for comparisons of the American hybrid strain with the European E- and Z- strains and Mann-Whitney tests used as post-hoc comparisons between two groups. All tests were performed using SPSS v. 17 software.

To obtain quantitative values for the measurements made in two earlier studies [11,13], the E/Z ratios were estimated by digitizing published Figure 1B in reference [11], and Figure 5 in reference [13].

## List of Abbreviations Used

ECB: European Corn Borer; OSN: olfactory sensory neuron; CNS: central nervous system; AFLP: amplified fragment length polymorphism; OR: olfactory receptor; ANOVA: analysis of variance.

## Authors' contributions

SBO carried out electrophysiological analyses, data analysis, and drafted the manuscript. KS assisted with electrophysiological analyses. ATG and DGH conceived of the study, generated crosses, and assisted with data analysis and manuscript revision, TD assisted with methodology and manuscript revision, and BSH participated in the design of the study and manuscript revision. All authors read and approved the final manuscript.

## Supplementary Material

Additional file 1 **Single sensillum response amplitude ratios comparing the spike amplitude of the response to the E pheromone isomer and the Z for all sensilla in each *O. nubilalis *strain or cross**. A) Populations are ordered according to predicted genotype assuming sex linkage of the peripheral amplitude trait (Table 1). B) Sensilla are arranged from lowest to highest ratio for each group. Ratios are presented at ***x***-50% so that sensilla with equal amplitude responses to both isomers lie at 0%. Sensilla above 0 have a higher amplitude response to the E isomer while Sensilla below 0 have a higher amplitude response to Z. Letters in the corner of each graph show least significant difference groupings (***p***> 0.05) according to post-hoc LSD tests for individual (Table 1). Populations with different letters are significantly different according to LSD tests (***p ***< = 0.05), and populations with multiple letters are common to more than one group. Click here for file

Additional file 2 **Average amplitude ratio histograms for all *O. nubilalis *populations**. Frequency distributions of average sensillum amplitude ratios combined from each cross shown in Figure 2. Populations are listed as in Table 1 and Figure 2: 1) parent E 2) parent Z 3) F_1 _hybrid EZ 4) F_1 _hybrid ZE 5) backcross EZxE 6) backcross EZxZ 7) backcross ZExE 8) backcross ZExZ 9) backcross ExZE 10) backcross ZxZE. Click here for file
