# Supplementary figures and images for: Ostrinia revisited: Evidence for sex linkage in European Corn Borer Ostrinia nubilalis (Hubner) pheromone reception
Source: BMC Evol Biol. 2010 Sep 16;10:285. doi: 10.1186/1471-2148-10-285 (PMC2955028; doi:10.1186/1471-2148-10-285)

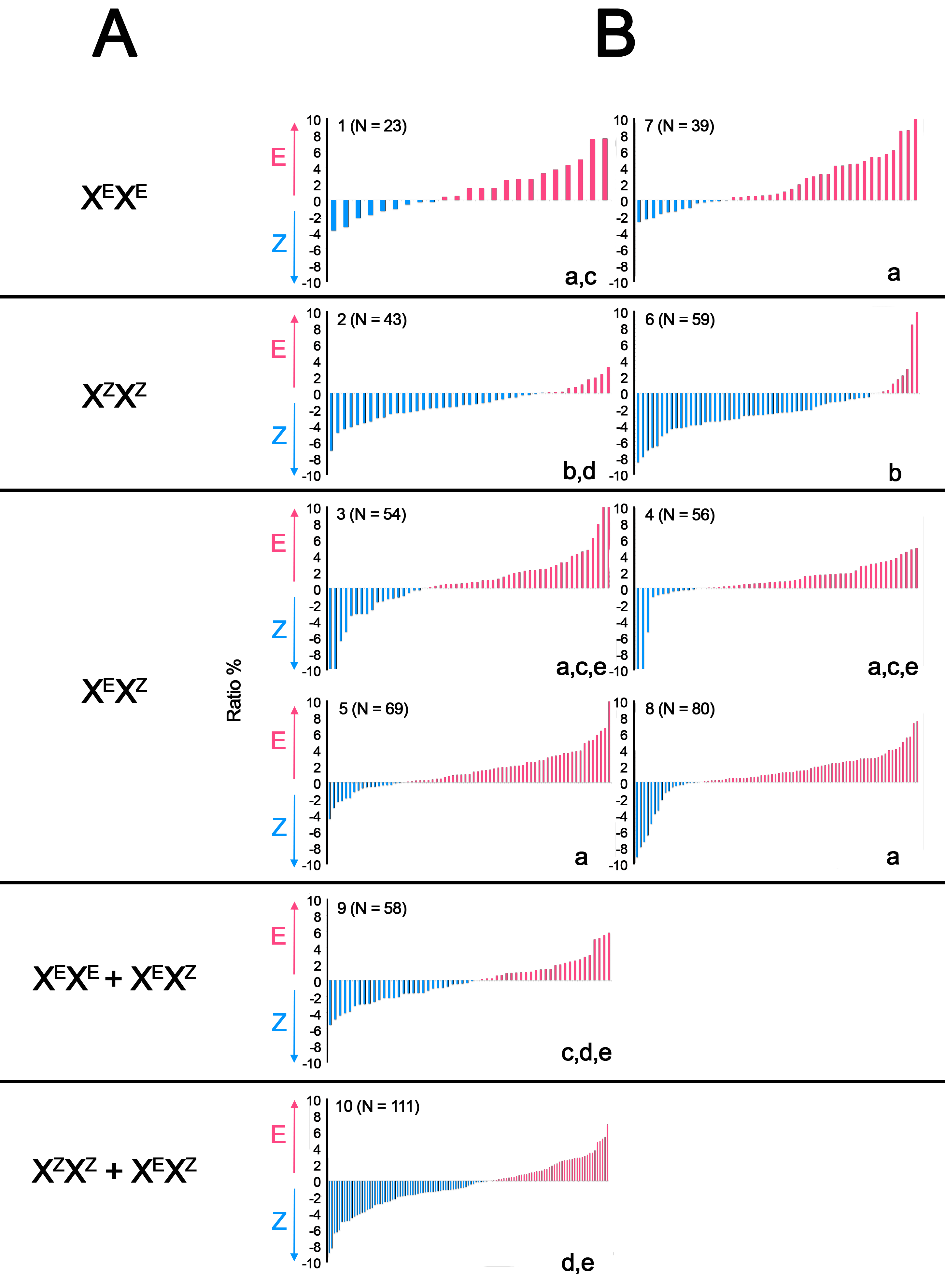

Supplement: Additional file 1 — Single sensillum response amplitude ratios comparing the spike amplitude of the response to the E pheromone isomer and the Z for all sensilla in each O. nubilalis strain or cross. A) Populations are ordered according to predicted genotype assuming sex linkage of the peripheral amplitude trait (Table 1). B) Sensilla are arranged from lowest to highest ratio for each group. Ratios are presented at x-50% so that sensilla with equal amplitude responses to both isomers lie at 0%. Sensilla above 0 have a higher amplitude response to the E isomer while Sensilla below 0 have a higher amplitude response to Z. Letters in the corner of each graph show least significant difference groupings (p> 0.05) according to post-hoc LSD tests for individual (Table 1). Populations with different letters are significantly different according to LSD tests (p < = 0.05), and populations with multiple letters are common to more than one group. [file 1471-2148-10-285-S1.PNG]

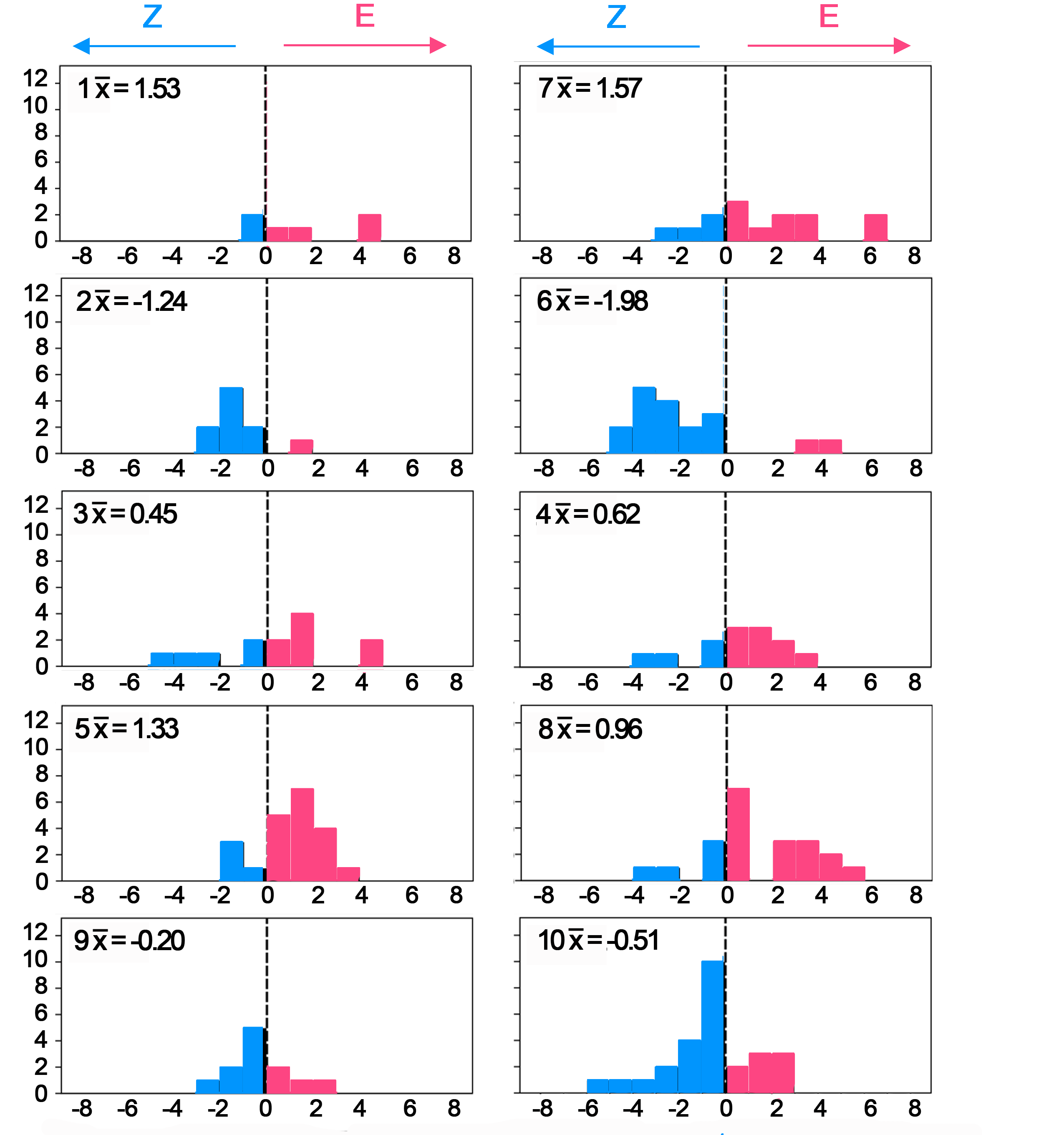

Supplement: Additional file 2 — Average amplitude ratio histograms for all O. nubilalis populations. Frequency distributions of average sensillum amplitude ratios combined from each cross shown in Figure 2. Populations are listed as in Table 1 and Figure 2: 1) parent E 2) parent Z 3) F1 hybrid EZ 4) F1 hybrid ZE 5) backcross EZxE 6) backcross EZxZ 7) backcross ZExE 8) backcross ZExZ 9) backcross ExZE 10) backcross ZxZE. [file 1471-2148-10-285-S2.PNG]
